# People in states worse than dead according to the EQ-5D UK value set: would they rather be dead?

**DOI:** 10.1007/s11136-018-1848-x

**Published:** 2018-04-03

**Authors:** Lars Bernfort, Björn Gerdle, Magnus Husberg, Lars-Åke Levin

**Affiliations:** 1 Division of Health Care Analysis, Department of Medical and Health Sciences, Linköping University, 58183 Linköping, Sweden; 2 Pain and Rehabilitation Centre, and Department of Medical and Health Sciences, Linköping University, Linköping, Sweden

**Keywords:** Patient-reported outcomes, Quality-adjusted life years, Life satisfaction, EQ-5D, Health state valuation, States worse than dead

## Abstract

**Purpose:**

Quality-adjusted life years (QALYs) measure health by combining length and quality of life. QALYs constitute the effect side of incremental cost-effectiveness ratios, describing the results of health economic evaluations. The objectives of this study were to (1) investigate the prevalence of states worse than dead (SWD) when using the EuroQol-5D UK value set, and (2) to study to what extent SWDs are reasonable with a starting point in experience-based valuations of health states.

**Methods:**

Data from a Swedish cross-sectional population survey were used. The survey was directed to 10,000 persons 65 years and older and its primary aim was to investigate the prevalence and consequences of chronic pain. The survey included questions reflecting life situation and well-being. Some of these were used in order to characterise people in SWD.

**Results:**

SWD were found in 1.8% of the 6611 respondents. The prevalence of SWD increased with advancing age and was more common among women than men. The control questions used indicated that most of the persons being in SWD according to the EQ-5D UK value set most probably would not judge themselves to be in a SWD.

**Conclusions:**

Though negative QALY-weights are not very common, they constitute a non-negligible part of health states in a Swedish population 65 years and older. Prevalence of SWD is higher among women than men and increases with age. From responses to other questions on well-being and life situation, there is reason to doubt the reasonableness of experience-based negative QALY-weights in many cases.

## Introduction

Quality-adjusted life years (QALYs) are used to measure health by combining the two central aspects of life: length and quality. Generic measures of quality of life generate QALY-weights on a scale with the anchor points zero that equals “dead” and one that equals “perfect health”. QALY-weights can be derived either by use of direct methods [1] such as standard gamble (SG) [2], time trade-off (TTO) [3] and visual analogue scale (VAS) [4], or by use of indirect methods, meaning that results of descriptive quality-of-life instruments are transformed into QALY-weights. A widely used indirect method for deriving QALY-weights is the EuroQol five-dimensional three-level (EQ-5D-3L) descriptive system [5], hereinafter referred to as EQ-5D, and a transformation into a value set of QALY-weights using a direct valuation method.

Value sets are used for calculating QALYs based on descriptive quality-of-life instruments such as the EQ-5D or the SF-36, which are commonly collected alongside clinical trials. QALYs for a specific state are calculated by multiplying the (QALY) weight attached to the state with the time spent in that state. As health states change over time, the total amount of QALYs for a person is the sum of the different states experienced (time in state × QALY-weight of state). Differences in QALYs between interventions being compared form one part of a cost–utility analysis (CUA), which is the most common form of health economic assessment. The difference in QALYs is related to the difference in costs between interventions. The result of a CUA comparing treatments A and B is presented in the form of an incremental cost-effectiveness ratio (ICER): (Cost_A_ − Cost_B_)/(QALYs_A_ − QALYs_B_). So, it is evident that the calculation of QALYs is of great importance for the resulting ICER.

The most widely used value set created from the EQ-5D is the UK value set [6, 7] that was constructed by using the TTO. In the UK value set, QALY-weights are allowed to take on negative values meaning that it is possible to be in a state that is valued as worse than being dead. About one-third of the 243 possible states from the EQ-5D were given negative values. The value set was created by letting 3395 representatives of the general population (2997 complete responses) value a subset of 45 states out of the 243 possible states. Each individual valued 13 hypothetical states one by one by use of the TTO technique. A TTO board was used with one side relevant for states better than dead and the other for states worse than dead. When valuing each health state, respondents were free to use the side of the board considered relevant. Valuations of the 45 states were interpolated to a value set for all 243 possible states. For states worse than dead, respondents were asked to state their preferences on the trade-off between a combination of time in the states “worse than dead” and “perfect health” on the one hand, and immediate death on the other. The point of indifference between the combination and immediate death determined how much worse than dead that the state was considered. The less time in the state worse than dead that could be endured, the more negative was the QALY-weight. Among the first to recognise the importance of health states worse than dead (SWD) in the context of scarce resources and health economic evaluations were Rosser and Kind [8]. Since then different methods for measuring SWD, mainly based on the time trade-off, have been developed [9–11].

The EQ-5D UK value set is based on hypothetical valuations of health states, meaning that representatives of the general public were asked to state their beliefs on the quality of life associated with health states described to them. The alternative to using hypothetical valuations is to use experience-based valuations, meaning that the quality of life of a particular health state is valued by those experiencing, or having experienced the health state. In Sweden, for example, experience-based valuations are preferred [12]. Nevertheless, in the absence of a Swedish value set, the UK value set has been used in health economic assessments informing decision making in the Swedish health care system. Reasonable arguments for and against either hypothetical or experience-based valuations have been presented in the literature [13–19]. Advocates of hypothetical valuations often argue that as the health care sector is financed by taxpayers, health care activities should be based on the expectations and preferences of taxpayers (i.e. the general public). Advocates of experience-based valuations argue that the only ones who really know what it is like to be in a health state are those with experience of the health state. An interesting question is how persons in states associated with (hypothetical) negative QALY-weights experience their situation. Do persons in SWD perceive their lives as not worth living? If not, is it reasonable that persons living fairly satisfying lives are attached negative QALY-weights?

The purpose of this study was to determine the prevalence of states worse than dead in a Swedish sample 65 years and older, using the EQ-5D UK value set. A further aim was to investigate how people in states worse than dead apprehend their states in terms of well-being.

## Methods

This study is based on data from a Swedish cross-sectional population survey, which aimed to study the prevalence and consequences of chronic pain among people 65 years and older [20]. The study sample consisted of 10,000 subjects to whom postal questionnaires were sent. The study sample was divided into five age strata and 2000 questionnaires were sent out in each stratum: 65–69, 70–74, 75–79, 80–84 and 85 years and older. All persons in the sample were residents in either of the municipalities of Linköping or Norrköping, two nearby municipalities in the Southeast of Sweden. Both municipalities have about 150,000 inhabitants with a mix of people living in the cities and in villages.

Besides questions focusing on pain, the questionnaire included the EQ-5D and questions on, for instance, happiness and well-being [20].

Questions reflecting well-being or life situation (e.g. life in general, happiness) were used as a way to describe how persons in SWD apprehend their life situation. Some of these questions are included in the general well-being schedule [21] and some are inspired by research on happiness, in which attempts are made to measure ‘subjective well-being’[22].

The questions or statements specifically used for mirroring well-being or life situation in this study were worded in the following way:

- “In total, how do you think that your life is nowadays? Would you say that you are:” (happy/fairly happy/not so happy)
- “In general, my life is...” (1–6, where 1 = very unsatisfactory and 6 = very satisfactory).
- “How happy, satisfied or pleased have you been with your personal life during the last 4 weeks?” (1–6, where 1 = extremely happy—could not have been more satisfied and pleased and 6 = very dissatisfied).
- “In total, how satisfied are you with your life nowadays?” (0–10, where 0 = very dissatisfied and 10 = very satisfied).

### Statistics

All data analyses were performed with SPSS version IBM Statistics 23 (IBM Corp., Armonk, NY). The *p* value ≤ 0.05 was applied.

Descriptive statistics, by proportion or mean, were used to present relationships between SWD and other measures reflecting well-being or life situation.

## Results

**Fig. 1:**
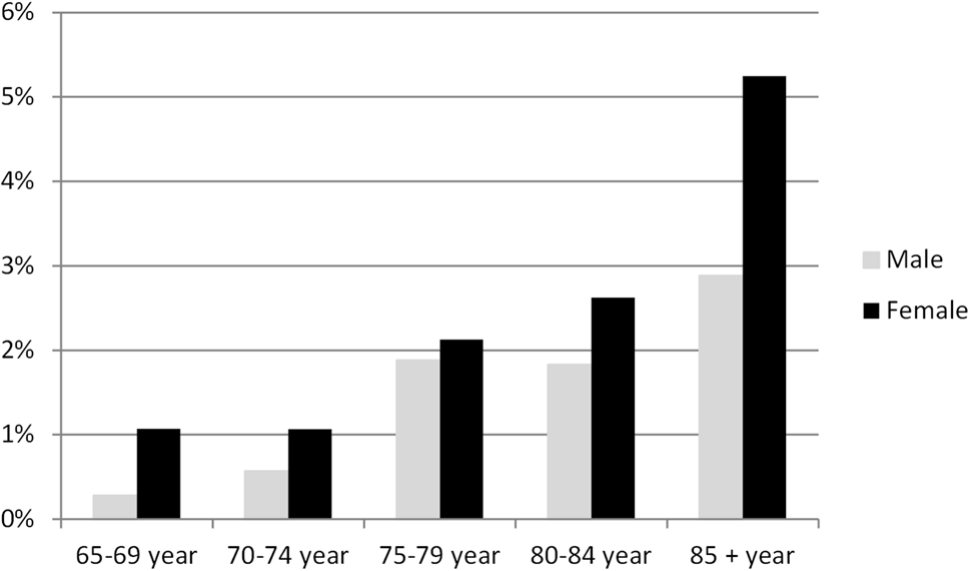
Proportions of persons in SWD in each age/sex group

The response rate to the survey was 66.5%. Due to internal loss, there were 6611 valid responses. Responders consisted of 54% women and the mean age was 76.2 years (range 65–102 years). The non-response analysis showed that response rates were higher among men than women, in the younger age strata, among married people, among those with high education and high income and for people born in Sweden compared to foreign born. The prevalence of SWD, according to the EQ-5D UK value set, was 1.8% for the whole study population. Table 1 and Fig. 1 give a general description of the study population, and the subpopulation in SWD, in relation to age and gender.

**Table 1:** Baseline characteristics for individuals in states better than dead (SBD) or states worse than dead (SWD)

	Better than dead (*n* = 6494)	Worse than dead (*n* = 115)	Statistics (*p* value)
Women (%)	53.4	67.0	0.004
Men (%)	46.6	33.0	
Age [years (mean)]	75.9	81.2	< 0.001
65–69 years (%)	23.2	8.7	
70–74 years (%)	23.1	10.4	
75–79 years (%)	21.1	23.5	
80–84 years (%)	18.7	23.5	
85 years and older (%)	13.8	33.9	

Table 1 shows that respondents in SWD were significantly older than respondents in SBD and the proportion of women was higher among respondents in SWD.

From Fig. 1 it is apparent that the prevalence of SWD increases with advancing age and is somewhat higher among women than men.

In order to investigate what characterise responders in SWD, comparisons were made with questions/measures reflecting the well-being and life situation of those people.

**Fig. 2:**
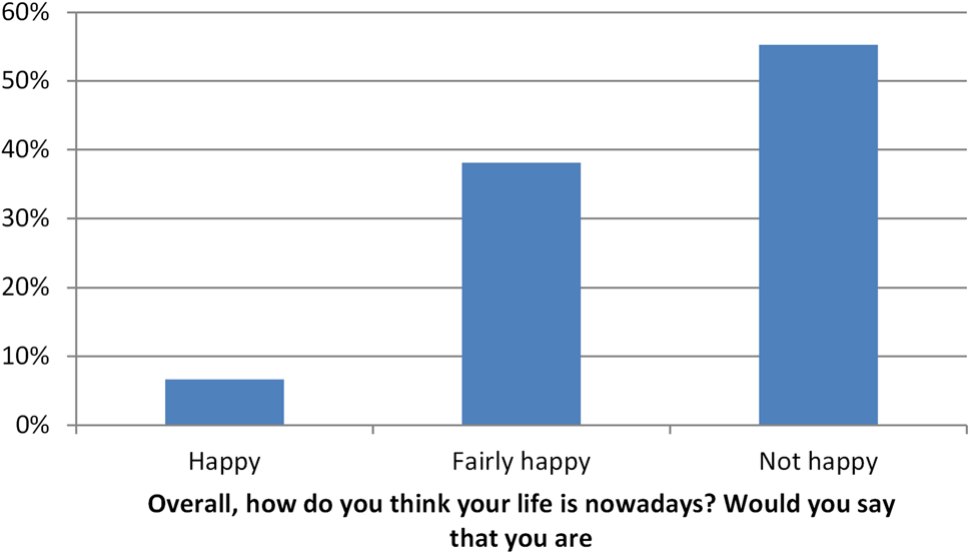
Responses by people in SWD to the question “Overall, how do you think your life is nowadays? Would you say that you are:”

The responses to this rather crude question concerning happiness indicated that about 55% of those in SWD were not happy (Fig. 2). However, what is interesting is that about 45% were fairly happy or even happy.

**Fig. 3:**
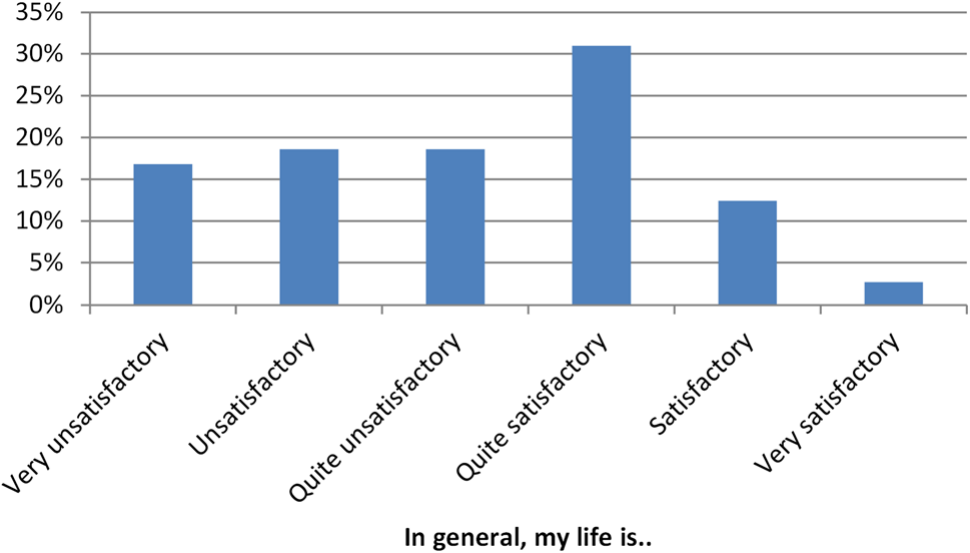
Responses by people in SWD to the statement “In general, my life is…”

Many of the persons that, according to the EQ-5D UK value set, were in states worse than dead unsurprisingly were not very satisfied with their life (Fig. 3). Somewhat surprisingly, as many as 46% of those in SWD expressed that their life was quite satisfactory, satisfactory or even very satisfactory.

**Fig. 4:**
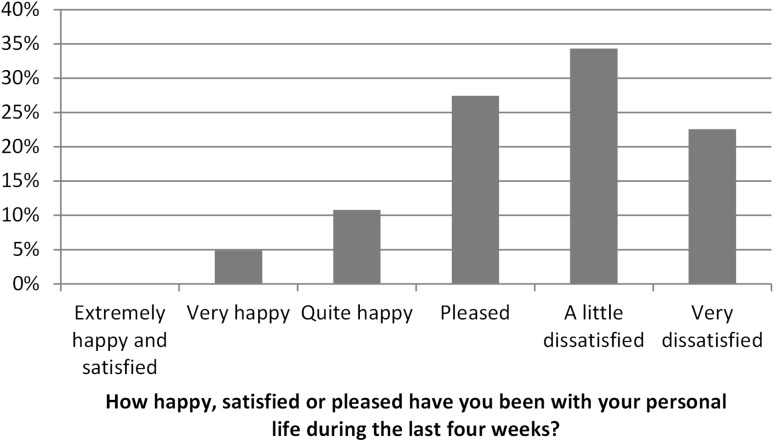
Responses by people in SWD to the question “How happy, satisfied or pleased have you been with your personal life during the last 4 weeks?”

When asking about the personal life during the last 4 weeks of persons in SWD, 57% were a little or very dissatisfied, and 43% were not dissatisfied (Fig. 4).

**Fig. 5:**
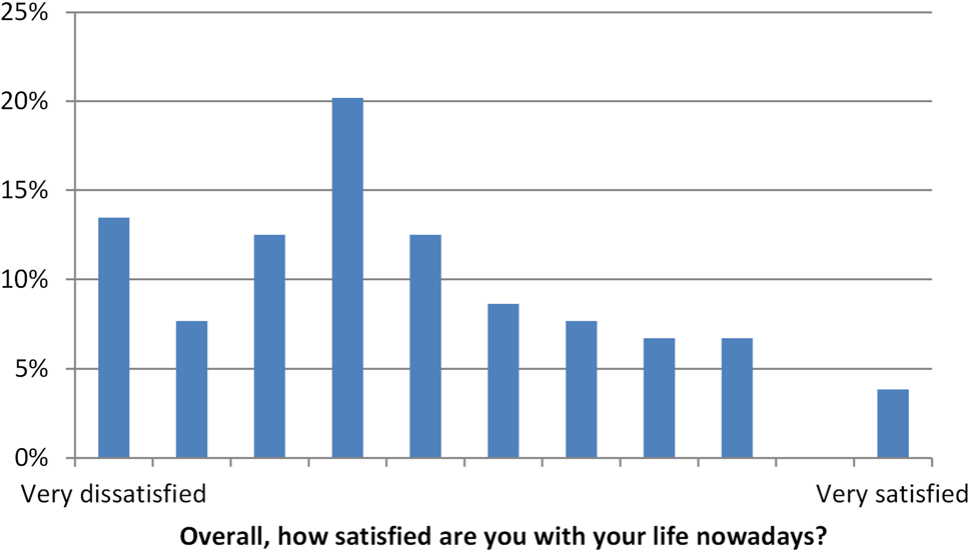
Responses by people in SWD to the question “Overall, how satisfied are you with your life nowadays?”

Also when analysing responses to the question concerning satisfaction it was found that most people in SWD were quite dissatisfied with their life, but about one-third of responders were fairly to very satisfied and less than 15% were very dissatisfied (Fig. 5).

As would be expected, people who were in states better than dead (SBD) had better outcomes to the questions on perception of life in general, happiness and satisfaction than people in SWD.

## Discussion

In this study we found that a non-negligible proportion of a Swedish general population of people 65 years and older were in states that according to the EQ-5D UK value set is considered to be worse than dead. We also found that a large proportion of the persons in SWD according to their own opinions experienced fair or even good well-being. These results make it reasonable to question the validity of the EQ-5D UK value set, at least in an elderly Swedish population.

In Swedish health economic evaluations, the EQ-5D UK value set has often been used for retrieving index values from the EQ-5D. This means that health state valuations that may not be valid have been used as basis for reimbursement decisions in Sweden. So, at least concerning an elderly Swedish population, previous and future decisions on reimbursements and resource allocation might be sub-optimal due to invalid valuations of health states. This fact disfavors development of functional care of the elderly. It is therefore important to undertake further research in order to find valid methods for valuing health states in a Swedish setting. Whether this is the case also for other populations in other countries needs to be investigated.

If people were really in states apprehended as worse than being dead, you would expect that (almost) all of them should be very unhappy and dissatisfied with their lives. Interestingly, however, a relatively large part of those in SWD report that they are not all that unhappy, dissatisfied or resigned. Ratings were relatively low, but it is difficult to conclude that most respondents’ health states were worse than dead.

A reason for comparing the EQ-5D value set with measures of self-reported general well-being is that in Sweden experience-based valuations are advocated. So, there has arguably been a profound discrepancy between guidelines and practice.

The discrepancies found might to some degree be explained by country-specific differences between Sweden and the UK, and the age distribution in our study population. The EQ-5D value set was not specifically validated for a population as old as ours, 65 years and older, and it might very well be that the view on quality of life differs between ages. What is considered severe limitations (e.g. problem walking) for young persons might be acceptable for old persons. The discrepancies might also depend on the fact that hypothetical valuations are different from experience-based valuations. Differences between the hypothetical valuations mirrored in the EQ-5D UK value set, and actual self-reported and self-experienced life satisfaction probably depend on the fact that people that are actually in poor health states tend to cope with their situation [23–26]. Hence, the EQ-5D UK value set is based on the anticipations of representatives of the general population; this approach may reasonably be sensitive to both knowledge and contextual factors, such as age and socioeconomic status.

Differences found between women and men, with more women judged to have very poor quality of life, are in line with previous research. Despite having a distinct advantage in survival, women above a certain age are consistently reported to have poorer quality of life than same-aged men [27–29]. An Austrian study [30] of people aged 57–95 found that below the age of 70 years women had better quality of life than men, but above the age of 70 the opposite was true. In our study, differences were most profound in the oldest age group (85+), containing a larger share of women as more of the men had died. Gender differences in quality of life above a certain age might be explained by women having on average poorer socioeconomic status than men. It might also be explained by the fact that more women were widowed and living alone. Socioeconomic status, close relations and social support are important determinants for quality of life [31, 32].

The fact that a non-negligible proportion of respondents in our study were found to be in SWD might be explained by the methods used when creating the EQ-5D UK value set. The fact that the states valued by respondents when creating the value set were hypothetical in combination with the method used for valuing SWD might spur respondents to value more states than reasonable as SWD. A more recently proposed method for valuing states worse than dead is the lead-time trade-off method [10], which mitigates the value problems of the standard TTO and would probably alter the EQ-5D UK value set.

Lead-time trade-off was applied in the recently developed value set for the five level version of EQ-5D (EQ-5D-5L) [33]. This value set is more in line with what our results suggest, in the sense that only 4.93% of the 3125 possible states are worse than dead and the most negative value attainable is − 0.281 (compared to − 0.594 in the EQ-5D-3L value set) [34].

The discrepancy between hypothetical and experience-based valuations could be argued not to be a problem as the choice between hypothetical and experience-based valuations builds on a normative stance. It is important, however, to be aware of the diverse meanings and results of the two alternative approaches when deciding which one to use. Our results indicate that the choice of hypothetical or experience-based valuations of health states might have a big impact on ICERs resulting from health economic evaluations. It should be noted that our main aim was to provide a plain illustration of the experienced well-being of people reportedly in SWD. For that purpose we compared hypothetical preference values, i.e. the EQ-5D UK value set based on TTO, with self-reported experience-based apprehensions of well-being. In order to actually quantify the differences in quality of life and thereby in ICERs resulting from hypothetical and experience-based valuations, a preference-based measurement tool, such as the TTO, would have to be administered to those experiencing conditions reportedly corresponding to SWD. Future research could examine this issue, which includes challenges not least connected to inclusion of people in presumably very poor health states in such measurement activities.

Our study population consists of people 65 years and older in a Swedish setting. Previous research has shown profound differences in valuations of health states between countries [35] and our results are not necessarily generalisable to other populations in other countries.

There are diverse opinions on who should value health states, i.e. whether hypothetical or experience-based valuations should be used in health economic evaluations. In the UK and most other European countries hypothetical valuations are preferred, while in, for instance, Sweden experience-based valuations are preferred [36]. Hypothetical and experience-based valuations of health states have previously been shown to give rather pronounced differences in results, for instance, when comparing the EQ-5D UK value set with the not yet validated Swedish experience-based value set [37–43]. Experience-based value sets have been suggested based on its performance compared to hypothetical value sets, in both generic and disease-specific settings [44–46].

We found associations showing that persons with poor quality of life according to the EQ-5D UK value set are certainly worse-off also according to other measures. This in some sense validates the EQ-5D UK value set but raises a concern whether it might be wrongfully calibrated with respect to level.

Our study has limitations. Our study population is obviously rather specific, consisting of people 65 years and older in Sweden, which makes generalisability of our findings uncertain. Our findings consist of plain illustrations of how people in health states worse than dead according to the EQ-5D UK value set apprehend their life situation in terms of non-quantifiable representations of general well-being. The rather small number of people in SWD in combination with the characteristics of the measures of general well-being makes any meaningful computation of statistical correlations impossible. Based on our results, we cannot comment on the sizes of possible differences between hypothetical and experience-based valuations of health states. Therefore, we cannot quantify the magnitude of differences in resulting ICERs, merely stating that the choice of valuation method is probable to have an impact on ICERs resulting from health economic evaluations.

## Conclusions

Even though negative QALY-weights are not very common, they constitute a non-negligible part of health states in a Swedish population 65 years and older. Our findings indicate that far from all the people in SWD were dissatisfied with their lives. Therefore, we find it reasonable to question the use of the EQ-5D value set, and the inherent prevalence of states worse than dead, at least in a setting where experience-based valuations are advocated. The choice whether to use hypothetical or experience-based valuations of health states might have a big impact on ICERs resulting from health economic evaluations.

## References

[1] Torrance GW (1976). Social preferences for health states—empirical evaluation of 3 measurement techniques. Socio-Economic Planning Sciences.

[2] Von Neumann J, Morgenstern O (1944). Theory of games and economic behaviour.

[3] Torrance GW, Thomas WH, Sackett DL (1972). A utility maximization model for evaluation of health care programs. Health Services Research.

[4] Patrick DL, Bush JW, Chen MM (1973). Methods for measuring levels of well-being for a health status index. Health Services Research.

[5] EuroQol-Group (1990). EuroQol—a new facility for the measurement of health-related quality of life. Health Policy.

[6] Dolan, P. (1995). *A social tariff for EuroQol: results from a UK general population survey*. Working paper. University of York: Centre for Health Economics.

[7] Dolan P, Sutton M (1997). Mapping visual analogue scale health state valuations onto standard gamble and time trade-off values. Social Science Medicine.

[8] Rosser R, Kind P (1978). A scale of valuations of states of illness: Is there a social consensus?. International Journal of Epidemiology.

[9] Torrance GW (1982). Preferences for health states: A review of measurement methods. Mead Johnson Symposium on Perinatal and Developmental Medicine,.

[10] Robinson A, Spencer A (2006). Exploring challenges to TTO utilities: Valuing states worse than dead. Health Economics.

[11] Richardson, J., & Hawthorne, G. (2001). *Negative utility scores and evaluating the AQoL all worst health state*. Monash Working Paper. Monash University: Centre for health program evaluation.

[12] TLV. (2003). *Guidelines for economic evaluations*. Retrieved April 4, 2016, from http://www.tlv.se/Upload/English/Guidelines-for-economic-evaluations-LFNAR-2003-2.pdf.

[13] Hadorn D (1991). The role of public values in setting health care priorities. Social Science Medicine.

[14] Brazier J, Akehurst R, Brennan A (2005). Should patients have a greater role in the valuation of health status?. Applied Health Economics and Health Policy.

[15] De Wit GA, Busschbach JJ, De Charro FT (2000). Sensitivity and perspective in the valuation of health status: Whose values count?. Health Economics.

[16] Ubel PA, Loewenstein G, Jepson C (2003). Whose quality of life? A commentary exploring discrepancies between health state evaluations of patients and the general public. Quality of Life Research.

[17] Dolan P (2008). Developing methods that really do value the ‘Q’ in the QALY. Health Economics Policy and Law.

[18] Weinstein MC, Siegel JE, Gold MR, Kamlet MS, Russell LB (1996). Recommendations of the panel on cost-effectiveness in health and medicine. JAMA.

[19] Dolan P, Kahneman D (2008). Interpretations of utility and their implications for the valuation of health. Economic Journal.

[20] Bernfort L, Gerdle B, Rahmqvist M, Husberg M, Levin LA (2015). Severity of chronic pain in an elderly population in Sweden—impact on costs and quality of life. Pain.

[21] Dupuy HJ, McDowell I, Newell C (1977). The General Well-being schedule. Measuring health: A guide to rating scales and questionnaire.

[22] Andrews F, Robinson J, Robinson J, Shaver P, Wrightsman L (1991). Measures of subjective well-being. Measures of personality and social psychological attitudes.

[23] Howell R, Kern M, Lyubomirsky S (2007). Health benefits: Meta-analytically determining the impact of well-being on objective health outcomes. Health Psychology Review.

[24] Brickman P, Coates D, Janoff-Bulman R (1978). Lottery winners and accident victims: Is happiness relative?. Journal of Personality and Social Psychology.

[25] Tyc VL (1992). Psychosocial adaptation of children and adolescents with limb deficiencies—A review. Clinical Psychology Review.

[26] Wu S (2001). Adapting to heart conditions: A test of the hedonic treadmill. Journal of Health Economics.

[27] Pinquart M, Sorensen S (2001). Gender differences in self-concept and psychological well-being in old age: A meta-analysis. Journals of Gerontology Series B-Psychological Sciences and Social Sciences.

[28] Orfila F, Ferrer M, Lamarca R, Tebe C, Domingo-Salvany A, Alonso J (2006). Gender differences in health-related quality of life among the elderly: The role of objective functional capacity and chronic conditions. Social Science & Medicine.

[29] Liang J, Bennett JM, Sugisawa H, Kobayashi E, Fukaya T (2003). Gender differences in old age mortality—roles of health behavior and baseline health status. Journal of Clinical Epidemiology.

[30] Kirchengast S, Haslinger B (2008). Gender differences in health-related quality of life among healthy aged and old-aged Austrians: Cross-sectional analysis. Gender Medicine.

[31] Bradbeer M, Helme RD, Yong HH, Kendig HL, Gibson SJ (2003). Widowhood and other demographic associations of pain in independent older people. Clinical Journal of Pain.

[32] Cheng ST, Chan ACM (2006). Relationship with others and life satisfaction in later life: Do gender and widowhood make a difference?. Journals of Gerontology Series B-Psychological Sciences and Social Sciences.

[33] Herdman M, Gudex C, Lloyd A, Janssen M, Kind P, Parkin D (2011). Development and preliminary testing of the new five-level version of EQ-5D (EQ-5D-5L). Quality of Life Research.

[34] Devlin N, Shah K, Feng Y, Mulhern B, van Hout B (2016). Valuing health-related quality of life: An EQ-5D-5L value set for England. Health Economics.

[35] Heijink R, Reitmeir P, Leidl R (2017). International comparison of experience-based health state values at the population level. Health and Quality of Life Outcomes.

[36] Heintz E, Gerber-Grote A, Ghabri S, Hamers FF, Rupel VP, Slabe-Erker R (2016). Is there a European view on health economic evaluations? Results from a synopsis of methodological guidelines used in the EUnetHTA partner countries. Pharmacoeconomics.

[37] Polsky D, Willke R, Scott K, Schulman K, Glick H (2001). A comparison of scoring weights for the EuroQol derived from patients and the general public. Health Economics.

[38] Peeters Y, Stiggelbout AM (2010). Health state valuations of patients and the general public analytically compared: A meta-analytical comparison of patient and population health state utilities. Value in Health.

[39] Little M, Reitmeir P, Peters A, Leidl R (2014). The impact of differences between patient and general population EQ-5D values on the mean tariff scores of different patient groups. Value in Health.

[40] Aronsson M, Husberg M, Kalkan A, Eckard N, Alwin J (2014). Differences between hypothetical and experience-based value sets for Eq-5d: Implications for decision makers. Value in Health.

[41] Kiadaliri AA, Eliasson B, Gerdtham UG (2015). Does the choice of EQ-5D tariff matter? A comparison of the Swedish EQ-5D-3L index score with UK, US, Germany and Denmark among type 2 diabetes patients. Health and Quality of Life Outcomes.

[42] Gulfe A, Wallman JK, Kristensen LE (2016). EuroQol-5 dimensions utility gain according to British and Swedish preference sets in rheumatoid arthritis treated with abatacept, rituximab, tocilizumab, or tumour necrosis factor inhibitors: A prospective cohort study from southern Sweden. Arthritis Research & Therapy.

[43] Burstrom K, Sun S, Gerdtham UG, Henriksson M, Johannesson M, Levin LA (2014). Swedish experience-based value sets for EQ-5D health states. Quality of Life Research.

[44] Leidl R, Reitmeir P (2011). A value set for the EQ-5D based on experienced health states: Development and testing for the German population. Pharmacoeconomics.

[45] Leidl R, Reitmeir P, Konig HH, Stark R (2012). The performance of a value set for the EQ-5D based on experienced health states in patients with inflammatory bowel disease. Value in Health.

[46] Leidl R, Schweikert B, Hahmann H, Steinacker JM, Reitmeir P (2016). Assessing quality of life in a clinical study on heart rehabilitation patients: How well do value sets based on given or experienced health states reflect patients’ valuations?. Health and Quality of Life Outcomes.

